# Analysis of High-Field-Induced Processes with Enthalpy Release in Martensite–Austenite MnCo(Fe)(GeSi) Alloys: Solving PPMS Artifact and Recovery of Heat Capacity

**DOI:** 10.3390/ma19061253

**Published:** 2026-03-22

**Authors:** Antonio Vidal-Crespo, F. Javier Romero, Jhon J. Ipus, Javier S. Blázquez

**Affiliations:** Departmento de Física de la Materia Condensada, ICMSE-CSIC, Universidad de Sevilla, P.O. Box 1065, 41080 Sevilla, Spain; avcrespo@us.es (A.V.-C.); fjromero@us.es (F.J.R.); jhonipus@us.es (J.J.I.)

**Keywords:** Physical Property Measurement System (PPMS), heat capacity, latent heat, magnetic-field-induced transitions, martensite alloys

## Abstract

**Highlights:**

**What are the main findings?**

- Analysis of an apparent anomaly detected by PPMS and reinterpretation of results.
- High-magnetic-field-induced transformation was detected, implying a subtle enthalpy (ΔH) contribution.
- Recovery of heat capacity data and estimation of ΔH.

**What are the implications of the main findings?**

- Modeling ΔH contribution during data acquisition yields physically sound results.
- Good interpretation of measurements allows for estimation of ΔH contributions.
- Checking the conductance of wires for good behavior is needed.

**Abstract:**

The relaxation calorimeter option in the commercial Physical Property Measurement System (PPMS) has become widely used. Since its introduction, the capabilities of this technique for specific heat measurements have been critically discussed, particularly to avoid misinterpretation of data near phase transitions. Traditional methods rely on cooling curves after sample excitation, where sharp latent heat contributions during heating lead to clear deviations from the fitting model. However, subtle but extended enthalpy contributions (e.g., strain release) may mask these effects, allowing both heating and cooling curves to be well fitted using the standard PPMS protocol. In this work, we develop a procedure that assumes a constant extra power supplied due to subtle enthalpy contributions, enabling consistent interpretation of both heating and cooling curves. This procedure allows: (1) correction of specific heat measurements; and (2) quantification of the enthalpy involved in the transition. The procedure is applied to a magnetic-field-induced transformation in MnCo(Fe)Ge(Si) alloys. Two samples were studied: a single-phase austenite without any field-induced transition, used as a reference, and a mixed austenite-martensite sample, in which apparent deviations in the conductance of the wires evidence the presence of the anomaly.

## 1. Introduction

Since the development of the Physical Property Measurement System (PPMS) from Quantum Design, this device has been adopted by many laboratories due to its versatility and its ability to control temperature and magnetic field over a broad range. Among these capabilities, the heat capacity option uses a thermal relaxation calorimeter that provides high precision for second-order phase transitions (SOPT). A review of the standard methods used for heat capacity measurements in PPMS can be found in [1].

However, the standard fitting of heating and cooling branches (obtained when the temperature of the sample is modified by a heat pulse) is not suitable for first-order phase transitions (FOPT) [2]. This is due to the presence of latent heat or other enthalpy contributions that violate the assumptions of the PPMS fitting protocol. The protocol presumes (1) a constant specific heat, $c_{P}$, within the temperature interval associated with each data point acquisition, and (2) that the only heat source is the power, P, applied to the dissipator during the measurement process. Close to a transition, the assumption of constant specific heat breaks down and, for first-order transitions, latent heat violates the second condition.

Different strategies have been implemented to study sharp second-order or first-order phase transitions. Shortly after the introduction of PPMS, Lashley et al. [2] analyzed the log-log plot of the cooling branch within the temperature range where the latent heat effects can be neglected. The scanning method analyzes the temperature relaxation data point-by-point [3]. The single pulse method utilizes a thermal pulse wide enough to drive the system across the full transition [4], yet it remains inapplicable for transitions spanning many degrees. Alternatively, small temperature increments (below 2 K) can be applied at separated intervals, combined with long stabilization times to ensure that consecutive pulses do not overlap in temperature [5]. More recently, Hanggai et al. [6], taking advantage of the thermal hysteresis of the first-order transition in MnFePSi alloys, designed a protocol using successive heat pulses to separate reversible heat capacity from latent heat.

As a result, within the advanced PPMS user community, there is growing awareness of the risks associated with using raw PPMS outputs without a critical analysis of possible extra heat sources [1].

In this work, we present the detection and interpretation of an apparent anomaly in specific heat data as obtained from PPMS in the MnCo_0.8_Fe_0.2_Ge_0.4_Si_0.6_ system in which austenite (P6_3_/mmc) and martensite (Pnma) phases coexist and give rise to two magnetic transitions at 275 and 335 K [7]. Determination of specific heat in these systems with interesting magnetocaloric properties is particularly useful to obtain the hardly measurable adiabatic temperature change from the easier isothermal magnetic entropy change measurements generally acquired from the Maxwell equation applied to isothermal magnetization curves.

## 2. Materials and Methods

Amorphous MnCo_0.8_Fe_0.2_Ge_1−x_Si_x_ with x = 0.4 and 0.6 were produced by mechanical alloying from a mixture of high-purity powders (>99%). A mass of 5 g of powders was milled in a Fritsch Pulverisette Vario (Fritsch, Idar-Oberstein, Germany) using steel balls and hardened steel vials of 80 cm^3^ at 250 rpm (disk/vial ratio −2) for 100 h with 10:1 ball-to-powder mass ratio. On heating, these amorphous systems exothermically crystallize.

**Figure 1:**
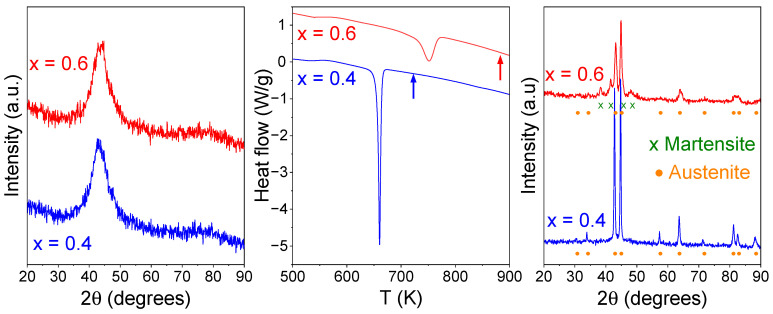
**Left** panel: XRD patterns of as-milled amorphous samples. **Center** panel: DSC scans at 20 K/min of as-milled samples. **Right** panel: XRD patterns of samples heated up to the temperature marked in the center panel by the corresponding arrow.

X-ray diffraction (XRD) experiments were performed using a Bruker D8 Advance A25 (Bruker, Karslruhe, Germany), Bragg–Brentano geometry, using the wavelength of Cu Kα. Figure 1 shows the XRD patterns of the as-milled and annealed systems. Both as-milled samples are homogenous amorphous systems; after crystallization, single-phase austenite is obtained for the x = 0.4 sample, whereas the x = 0.6 sample crystallizes in a biphasic mixture of austenite and martensite phases. Details on microstructure evolution with milling and composition can be found in [7].

Amorphous powders were pressed into 5 mm diameter disks using a uniaxial press with an applied load of 20 kN to facilitate sample handling. These disks were subsequently heated above the crystallization temperature using a Perkin-Elmer DSC7 power-compensated differential scanning calorimeter (Perkin-Elmer, Shelton, CT, USA). Pieces of these crystalline samples with masses 56.38(1) mg and 15.73(1) mg for x = 0.4 and 0.6, respectively, were mounted in a Physical Properties Measurement System (PPMS) from Quantum Design (Quantum Design, San Diego, CA, USA) equipped with a heat capacity measurement puck, which operates up to 400 K and 9 T. Before mounting each sample, the signal corresponding to the addenda was measured. The addenda include the Apiezon H grease (Apiezon, Salford, UK) used to stick the sample to the sample holder. Thermal contact between samples and holder was above 98%.

Heat capacity measurements were carried out using constant power pulses automatically selected by the PPMS to produce a temperature rise of approximately ΔT ≈ 2 K. The PPMS software (MultiVu 1.5.11, Heat Capacity Option 3.9.6) simultaneously fitted ΔT(t) heating and cooling branches through its standard relaxation routine, providing the characteristic relaxation time, τ, and the wire conductance parameter, $K_{1}$. These fitted parameters were monitored for consistency at all temperatures and magnetic fields, and selected data points were repeated to confirm reproducibility.

Magnetization measurements were performed in the vibrating sample magnetometer device of PPMS in the same sample used for calorimetry measurements.

## 3. Results and Discussion

**Figure 2:**
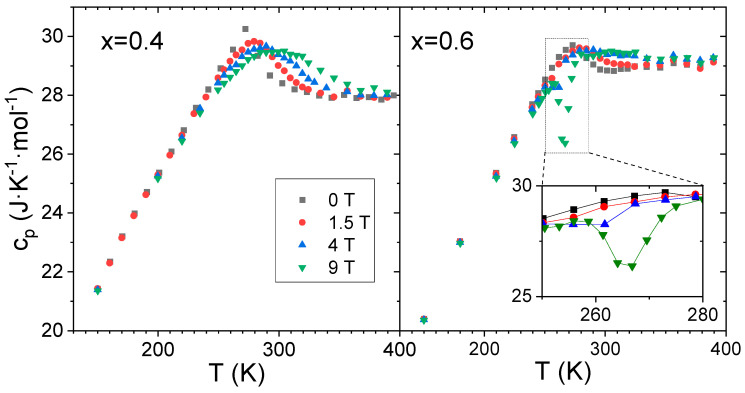
Specific heat measurements directly supplied by PPMS fitting software. The inset in the x = 0.6 curve corresponds to a zoom-in of the framed area.

Heat capacity, $C_{P}^{s}$, measurements as supplied from the PPMS are shown in Figure 2 for both crystallized samples at different magnetic fields. The sample with x = 0.4 shows a single and broad peak ascribed to the Curie temperature of the austenite phase at 277 K [7]. This peak becomes increasingly smeared and shifts to higher temperatures as the magnetic field increases from 0 to 9 T.

This behavior, which is typically found in magnetic SOPT, departs from the predictions of mean field theory, which anticipates a field-independent peak temperature for pure systems. However, such deviations are expected when the critical exponents differ from the mean field values [8], or when a distribution of Curie temperatures is present in the material, even if mean field values would otherwise apply [9].

The sample with x = 0.6 shows, at low fields (below 4 T), a qualitatively similar behavior to that of x = 0.4, although the peak is broader. This broadening is consistent with the presence of a distribution of Curie temperatures around 285 K for the austenite phase and around 335 K for the martensite phase [7]. However, at magnetic fields ≥4 T, a sharp decrease appears in the $C_{P}^{s}$ values directly supplied by PPMS at 260 K. This fall becomes more pronounced at 9 T, and the expected values of $C_{P}^{s}$ are recovered above 280 K.

Figure 2 corresponds to measurements cooling from 400 K. It is worth mentioning that this feature has been confirmed after several experiments, including both heating and cooling and sweeping of temperature for data acquisition. Moreover, thermal contact was not affected and remained during all the measurements at high values above 98%. Such peaks in $C_{P}^{s}$ are abnormal and, in fact, this anomaly will be shown below to correspond to an artifact due to the procedure followed in PPMS to obtain heat capacity values.

As explained in the Materials and Methods Section, the PPMS fitting routine provides τ and $K_{1}$ for each pulse during the acquisition of a single datum. To interpret the anomaly observed in the x = 0.6 sample at high fields, we now summarize the underlying relaxation formalism and introduce the working expressions used in our analysis. As a relaxation calorimeter, the PPMS device supplies a constant power $P_{0}$ for a short time $t_{0}$ to obtain $C_{P}^{s}$ at a certain temperature that yields a temperature rise in the system. Once the power is switched off, for $t>t_{0}$, the system temperature decreases. Therefore, the PPMS software fits the heating and cooling responses, even when the temperature rise is far from saturation. Both temperature rise and decrease depend on the heat capacity of the system as described by the following differential equations [3]:

(1) $$C_{P}^{p}\frac{dT_{p}}{dt}=P-K_{2}\left(T_{p}-T_{s}\right)-K_{1}\left(T_{p}-T_{b}\right)$$

(2) $$C_{P}^{s}\frac{dT_{s}}{dt}=K_{2}\left(T_{p}-T_{s}\right)$$

where $C_{P}^{s}$ is the heat capacity of the sample and $C_{P}^{p}$ is the heat capacity of the platform, thermometer, heater and thermal grease, i.e., the addenda. $K_{1}$ is the thermal conductance between the platform (at temperature $T_{p}$) and the heat sink (at $T_{b}$); and $K_{2}$ is the thermal conductance between the sample (at temperature $T_{s}$) and the platform to which it is attached; P is the power supplied during the heating pulse. When thermal coupling between sample and platform is good enough (in our case we had better than 98% coupling in all the experiments), the approximation $T_{p}=T_{s}$ is valid. Under this condition, the equations above can be simplified so that they depend only on $K_{1}$, yielding the following solution for the temperature evolution while P is applied:

(3) $$T_{s}\left(t\right)=T_{b}+\Delta T(1-e^{-\frac{t}{\tau }})$$

Analogously, when the power supplied is removed, sample and platform temperatures decrease following the equation:

(4) $$T_{s}\left(t\right)=T_{b}+\Delta T\left(1-e^{-t_{0}/\tau }\right)e^{-\left(t-t_{0}\right)/\tau }$$

This solution is valid for $t>t_{0}$, where $t_{0}$ represents the time at which the power supplied P is removed. When this approximation is not valid, the effect of $K_{2}$ must be explicitly taken into account [3]. Even a triple exponential decay is considered by Matsumoto et al. [10] for very low temperatures when thermal relaxation between nuclear and electron contributions applies.

**Figure 3:**
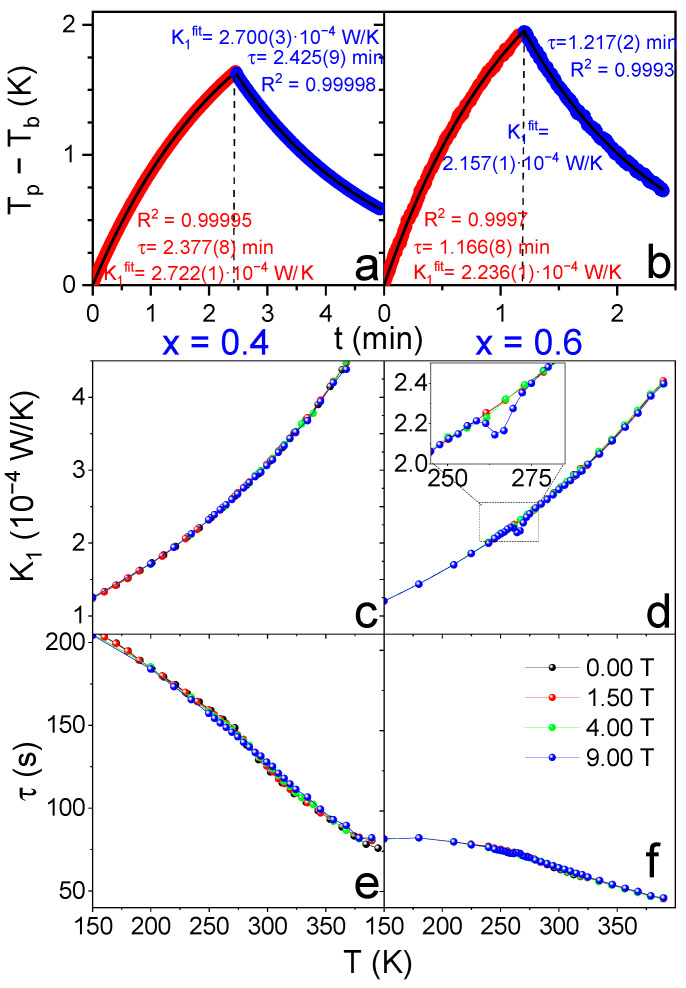
Heating and cooling curves obtained in the PPMS after applying and removing $P_{0}$ along with the fitting parameters obtained by applying Equations (3) and (4) for two examples: (**a**) x = 0.4, $T_{b}=271\;K,\;B_{app}=0\;T,\;P_{0}=6.919\cdot 10^{-4}\;W$ and (**b**) x = 0.6, $T_{b}=266\;K,\;B_{app}=9\;T,\;P_{0}=6.800\cdot 10^{-4}\;W$. Fitted values of conductance of the wires connecting the platform to the heat sink as supplied by the PPMS, $K_{1}^{fit}$, for x = 0.4 (**c**) and x = 0.6 (**d**). Relaxation time, τ, as fitted by the PPMS for x = 0.4 (**e**) and x = 0.6 (**f**).

Figure 3a,b shows examples of heating and cooling curves from individual data point acquisition events of the PPMS for two representative cases: (left) at 271 K and $B_{appl}=$ 0 T for the x = 0.4 sample, for which no anomaly is observed; and (right) at the data point where the anomaly is maximum among all heat capacity data obtained in this study (x = 0.6, T = 265 K and $B_{appl}=$ 9 T). These experimental curves can be fitted with Equations (3) and (4) with no apparent indication of an improper measurement.

Consequently, the PPMS software provides, without issuing any warning, the fitted parameters τ and ΔT, from which $K_{1}^{fit}=P_{0}/\Delta T$ and $C_{P}^{s}=K_{1}^{fit}\tau -C_{P}^{add}$, where $C_{P}^{s}$ is the heat capacity of the sample and $C_{P}^{add}$ the heat capacity of the addenda that was obtained in a previous measurement without the sample. In general, users directly access the computed $C_{P}^{s}$ values supplied by the software.

As evidenced in Figure 3b, the fitting procedure is adequate, and thus strong deviations from the assumptions of PPMS measurements as observed in FOPT are not expected. Therefore, unlike FOPT studied by Lashley et al. [2] and Hanggai et al. [6], no change in the slope of the log-log plot is associated with the anomaly detected in $C_{P}^{s}$. This indicates that the underlying transition is subtler in character and extended over the full thermal excitation imposed during the measurement.

In fact, when the raw data of the PPMS are examined in more detail, the temperature and field dependences of $K_{1}^{fit}$ and τ, shown in Figure 3 for x = 0.4 (c, e) and x = 0.6 (d, f), reveal deviations from regular behavior, particularly in the case of $K_{1}^{fit}$. These data are supplied by PPMS software and assume a constant $P_{0}$ and $t_{0}$, which are adjusted at each temperature to obtain a temperature rise of ΔT=2 K (value sets in our experiments).

In the case of x = 0.4, as temperature increases, a monotonous increase in $K_{1}^{fit}$ and a monotonous decrease in τ are observed, with both quantities remaining independent of magnetic field. This is also the behavior observed for x = 0.6 out of the temperature interval where the anomaly in $C_{P}^{s}$ is detected. However, within the range from 260 to 280 K, clear deviations in $K_{1}^{fit}$ emerge for $B_{app}=$ 4 and 9 T, while noise increases in τ but within the error range of the measurements. Scheie [11] also analyzed the behavior of $K_{1}^{fit}$ for quantitatively accurate results in the design of LongHCPulse software.

There is no physical reason for $K_{1}$, which corresponds to the thermal conductance of the wires connecting the platform to the heat sink, to depend on the applied magnetic field or to change in this temperature range for x = 0.6 but not for the x = 0.4 sample. We therefore attribute this apparent change in $K_{1}^{fit}$ to a misinterpretation of the conditions of the measurement by PPMS fitting routines in the case of the x = 0.6 sample.

In fact, the fitted parameters in Equations (3) and (4) are τ and ΔT, which are subsequently interpreted assuming a constant value of $P_{0}\sim 7\cdot 10^{-4}$ W. Under this assumption, $K_{1}^{fit}=P_{0}/\Delta T$, and thus $K_{1}^{fit}$ should be viewed strictly as a derived parameter from the PPMS software rather than a directly measured physical quantity.

**Figure 4:**
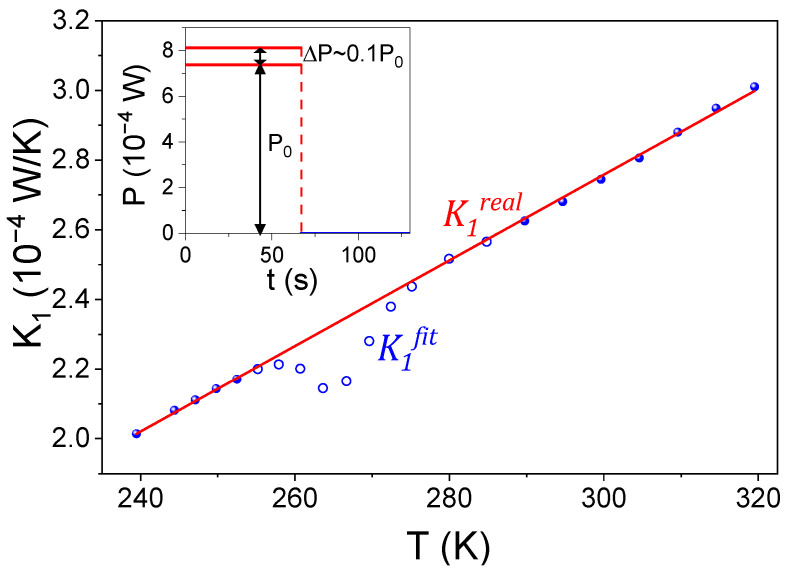
Values of $K_{1}^{real}$ (red line) and $K_{1}^{fit}$ (symbols) values. Open symbols correspond to those that deviate from the close-to-linear behavior in the region of the anomaly. Solid symbols are used to interpolate $K_{1}^{real}$ data in the region of the anomaly. The inset shows a scheme of our assumption of a constant extra power supplied due to the sample added to the constant $P_{0}$ supplied by the PPMS to understand the deviations between $K_{1}^{real}$ and $K_{1}^{fit}$ values.

Figure 4 shows the interpolated data for $K_{1}$ in the region of the anomaly (red line). These values, which we denote $K_{1}^{real}$, allow us to reinterpret the fitted ΔT values as $\Delta T=\left(P_{0}+\Delta P\right)/K_{1}^{real}$ assuming that during the heating process, an extra power ΔP was effectively supplied as a consequence of an enthalpy contribution originating in the sample itself, i.e., heat released ascribed to the process induced at high fields. We attribute this enthalpy release to the reorientation of martensitic variants under applied magnetic fields. As a first approximation, this ΔP is considered constant and restricted to the time interval $0\leq t\leq t_{0}$.

**Figure 5:**
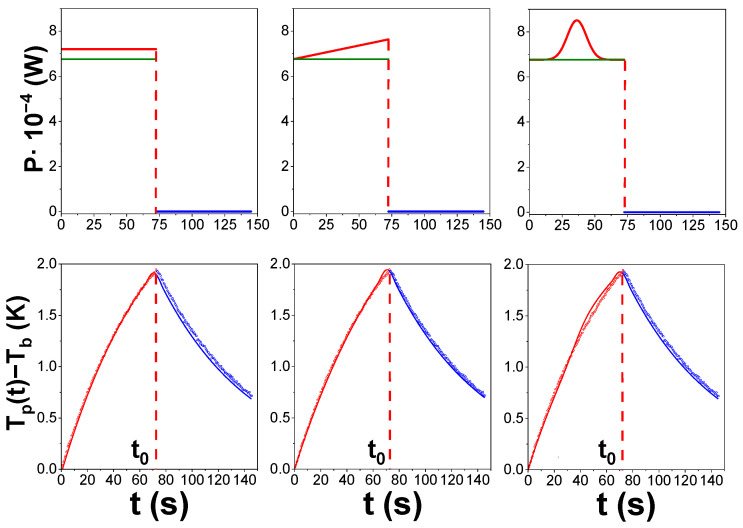
Effect of different profiles for extra power application on the quality of the fitting of a single datum acquisition in PPMS. Green lines in the upper row panels correspond to the power supplied by the equipment and red lines to the profiles for extra power.

Other extra power profiles were modeled to account for this sample contribution as an extra heating source during the excitation of the sample in data acquisition. Figure 5 shows the fitting of the heating excitation and cooling curves obtained. As no clear improvement is achieved, we chose the simplest approach, which is the constant ΔP during heating.

The PPMS relaxation method does not provide explicit standard deviations for the fitted parameters $K_{1}^{fit}$ and τ, but their uncertainties can be inferred from reproducibility and from the known limitations of the model used by PPMS. Typical PPMS fitting uncertainties are reported to lie in the 1–5% range, arising mainly from assumptions of constant heat capacity during a pulse and the exponential fitting procedure. According to the manufacturer’s specifications, the PPMS heat-capacity option typically reaches <2% accuracy under standard conditions, with a maximum quoted uncertainty of ±5% between 2 K and 300 K. Additional independent analyses of PPMS relaxation curves confirm that the percent-level scatter originates from the exponential thermodynamic model used in the fitting algorithm [11].

From repeated heating/cooling acquisitions in our measurements, we estimate uncertainties of ±0.5–1% in ΔT and ±2% in τ. Since the fitted conductance is obtained as $K_{1}^{fit}=P_{0}/\Delta T$, its uncertainty is dominated by ΔT, i.e., ±1–2%. The corrected conductance $K_{1}^{real}$, interpolated from the regular non-anomalous region, shows a point-to-point variability of ≈±1.5%. Therefore, the extra-power term ΔP carries a combined relative uncertainty of ≈±3%. Integration of ΔP(t) over the measurement interval leads to an estimated total enthalpy uncertainty of ≈±5–7%. These uncertainties are significantly smaller than the magnitude of the anomaly itself and do not affect the physical interpretation or the corrected heat capacity curves.

**Figure 6:**
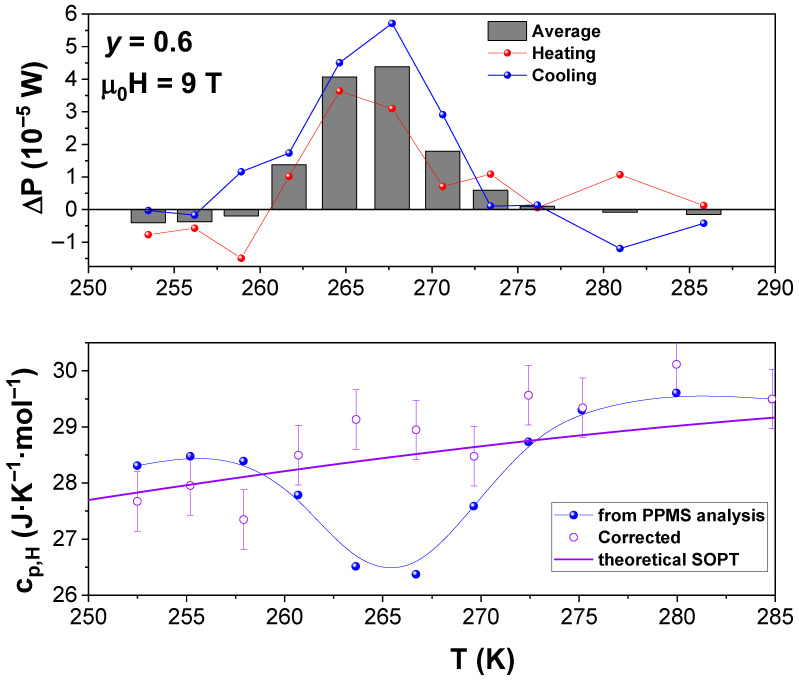
Upper panel: Histogram of the extra power supplied in the region of the anomaly obtained as the average values from the fitting of heating (red) and cooling (blue) curves at each data measurement. Lower panel: Corrected specific heat values (normalized to number of atoms instead of formula units) $c_{P}=C_{P}^{s}/n$, n=m/M, where M is the average atomic mass, (purple hollow symbols) along with those directly supplied by PPMS in the region of the anomaly (blue solid symbols). The purple line corresponds to the theoretical curve of $c_{P,9T}=c_{L}+c_{E}+c_{M,9T}$, where $c_{L}$ is the lattice contribution (Debye model, with Debye temperature $\theta_{D}=353\;K$, and $c_{E}$ is the electronic contribution with parameter $\gamma =8.44{\;\mathrm{mJ}}^{-1}K^{-2}$ (Data taken from [12]) and $c_{M,9T}$ is the magnetic contribution for which the response of x = 0.4 has been taken as standard response and normalized to $t=\left(T-T_{C}\right)/T_{C}$ to obtain individual contributions considering the histogram obtained in [7].

This interpretation allows us to correct the $C_{P}^{s}$ values from the artifact due to the extra enthalpy contribution from the sample using the corrected $K_{1}^{real}$ values in $C_{P}^{s}=K_{1}^{real}\tau -C_{P}^{add}$ and to build up a histogram with the values of $\Delta P=K_{1}^{real}\Delta T-P_{0}$ obtained at each temperature of measurement and ascribed to the possible martensitic variant reorientation process induced by the field. Both results are plotted in Figure 6. To validate the corrected heat capacity values, we constructed a theoretical $c_{P,9T}\left(T\right)$ curve for an applied field of 9 T, summing the lattice, electronic, and magnetic contributions. The lattice contribution $c_{L}$ was modeled using the Debye law with $\theta_{D}=353\;K$, while the electronic term $c_{E}=\gamma T$ employed $\gamma =8.44{\;\mathrm{mJ}}^{-1}K^{-2}$, both values taken from [12]. The magnetic contribution at 9 T, $c_{M,9T}$, was obtained from the behavior of the x = 0.4 sample after subtraction of the lattice and electronic contributions and subsequently rescaled according to the reduced temperature $t=\left(T-T_{C}\right)/T_{C}$. The weighting of magnetic contributions for the x = 0.6 composition was introduced through the histogram describing the distribution of Curie temperatures in this sample and previously determined in [7]. This procedure yields the theoretical reference curve shown as the purple line in Figure 6, to which the corrected experimental data can be directly compared.

From the resulting histogram of latent heat (Figure 6, upper panel), a $\Delta H\sim \left(\sum \Delta P\left(T_{i}\right)t_{0i}\right)/m\sim 3{\;\mathrm{kJ}\;\mathrm{kg}}^{-1}$ can be approached as an integral value. This value must be considered a low limit, due to two different sources for enthalpy losses during measurements: (1) the overshooting in temperature for thermal stabilization prior to data acquisition and (2) the difference between the ΔT achieved in the heating experiment (≤2 K) and the temperature gap between two consecutive data points (2.5 K in the range of the anomaly).

In the present case, since cooling and heating temperature sweeps yield identical results, the thermal hysteresis of the process must be negligibly small (unlike the observed case of Hanggai et al. [6]), and the overshooting issue is minimized. Therefore, we can estimate $\Delta H_{loss}\sim 0.25\Delta H$.

A transition from martensite to austenite phase induced by the field should not be consistent with the $C_{P}^{s}$ measured at temperatures above the anomaly, which is not affected by the magnetic field, and thus no change in the phase balance should be expected. Moreover, stabilization of the austenite phase with magnetic field is also incoherent with the lower Curie temperature of austenite with respect to martensite in this system.

**Figure 7:**
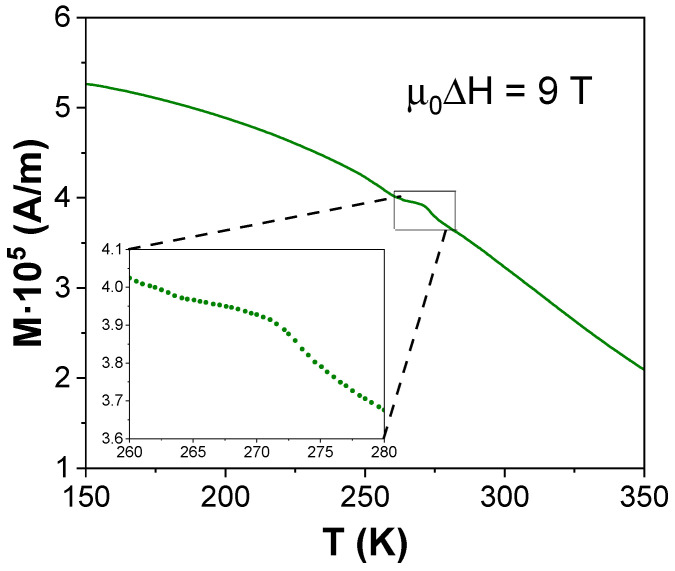
Magnetization measurements at 9 T for the x = 0.6 sample. The inset shows the individual experimental data points in a zoomed area.

The enthalpy contribution is only observed at very high fields. A plausible explanation is the reorientation of martensite variants, which are strongly anisotropic and therefore sensitive to the magnetic field in the ferromagnetic state (i.e., the temperature range of the anomaly). In situ microstructural observations are not feasible under the conditions of these measurements. However, magnetic-field-induced martensite variants rearrangement has been inferred from the variation in the thermal strain between zero field and 10 T for Ni(MnFe)Ga polycrystalline samples [13] affecting martensite variant orientation for a magnetic field of 0.75 T [14]. Temperature dependence of magnetization at 9 T for the x = 0.6 samples is shown in Figure 7 and shows a small increase in the temperature region where the artifact in $C_{P}^{s}$ is detected. This increase is in agreement with the proposed reorientation of martensite variants.

Reports of the energy released during magnetic-field-induced variant reorientation are scarce. To our knowledge, only Fukuda et al. [15] reported a value of ~200 kJ/m^3^ (~20 J/kg) for Fe-Pt alloys under the application of a 4 T field. In our case, for 4 T, the deviation of $K_{1}^{fit}$ from the interpolated data yields a single prominent point (Figure 3d, inset, green line), whose integration over the acquisition time results in an energy release of 30 J/kg, in fairly good agreement with the results from Fukuda.

Finally, the apparent anomaly detected in $C_{P}^{s}$ is, in fact, an artifact arising from the standard PPMS analysis routine, which assumes the absence of any additional enthalpy contribution and presumes a constant specific heat within the time window of each heat-pulse measurement.

## 4. Conclusions

A magnetic-field-induced process is observed in the martensitic MnCo_0.8_Fe_0.2_Ge_0.4_Si_0.6_ alloy, whereas no such effect appears in the MnCo_0.8_Fe_0.2_Ge_0.6_Si_0.4_ composition, which crystallizes as a single austenite phase. This process is likely associated with the high field reorientation of martensitic variants and leads to an artifact in the specific heat data directly supplied by the PPMS. By performing a simple analysis of the raw PPMS output and assuming a constant extra power contribution during the heating pulse, we reinterpret the relaxation data and estimate the enthalpy released during the magnetic-field-induced process. This approach also enables us to correct the specific heat values originally provided by the PPMS software.

## Data Availability

The original contributions presented in this study are included in the article. Further inquiries can be directed to the corresponding author.
